# A *Salmonella* phage steers bacterial populations toward hypersensitivity to benzalkonium chloride, hydrogen peroxide, and triclosan

**DOI:** 10.1128/aem.01437-26

**Published:** 2026-10-01

**Authors:** Shelyn Wongso, Philip Lauman, Lin Chen, Siyun Wang

**Affiliations:** 1 Food, Nutrition and Health, Faculty of Land and Food Systems, The University of British Columbia 117198, Vancouver, British Columbia, Canada; 2 School of Chemistry, Chemical Engineering and Biotechnology, Nanyang Technological University 541846, Singapore, Singapore; University of Georgia Center for Food Safety, Griffin, Georgia, USA

**Keywords:** phage biocontrol, phage steering, evolutionary trade-off, lipopolysaccharide, disinfectant, food safety, *Salmonella*

## Abstract

Phage steering is an emerging antimicrobial strategy that exploits the evolutionary consequences of phage predation to sensitize bacterial populations to existing antimicrobials. While phage steering toward hypersensitivity to antibiotics and immune effectors has been well documented, little is known about whether a similar mechanism can enhance bacterial sensitivity to commercial disinfectants. Here, we show that SF1, a lytic siphovirus infecting *Salmonella enterica*, requires an intact O-antigen layer for adsorption and lytic replication, and that in-frame deletion mutants with O-antigen or deeper LPS defects had increased susceptibility to several commercial disinfectants. Critically, SF1 enhanced the activity of all disinfectants tested in combination assays and, moreover, exhibited mathematical synergy with benzalkonium chloride, hydrogen peroxide, and triclosan, compounds to which SF1-selected LPS mutants were hypersensitive. These findings are consistent with the hypothesis that SF1 functions not only as a bactericidal agent, but as an evolutionary steering tool that potentiates sanitizer activity through selection for sanitizer-hypersensitive phenotypes. Broadly, these results support phage steering as a promising strategy for deploying bacteriophages as adjuvants to commercial disinfectants.

**IMPORTANCE:**

Commercial disinfectants are essential for controlling pathogens throughout the food production and processing chain, but their effectiveness is increasingly compromised by bacterial tolerance and persistence. Although bacteriophages are being investigated as direct bactericidal agents, their utility as adjuvants to commercial sanitizers remains underexplored. In this study, we demonstrate that *Salmonella* phage SF1 selects for LPS-deficient mutants that exhibit increased susceptibility to several commercial disinfectants. Importantly, we further show that SF1 synergizes with sanitizers to which phage-selected mutants are hypersensitive, supporting a steering-mediated mechanism whereby phage predation enhances sanitizer activity. To our knowledge, this is the first report demonstrating phage steering as a strategy for deploying bacteriophages as synergizing adjuvants to commercial disinfectants. These findings highlight the potential value of exploiting evolutionary trade-offs to improve pathogen control in food processing environments.

## INTRODUCTION

The widespread use of antimicrobial agents in food production and processing systems has selected for the emergence of resistant bacterial populations, challenging conventional approaches to pathogen control and motivating the development of complementary antibacterial strategies (1–4). One increasingly investigated approach is phage biocontrol (PB), which employs naturally occurring, highly specialized bactericidal viruses (bacteriophages) to infect and eliminate bacterial pathogens (5, 6). In particular, recent work has increasingly focused on phage steering, wherein bacteriophages are utilized as selective agents to bias bacterial populations toward more tractable phenotypes. In this context, phages simultaneously function as both bactericides and adjuvants that enhance the efficacy of existing antimicrobials (7, 8).

All phages belonging to the order *Caudoviricetes* are capable of lytic replication, in which viral amplification culminates in lysis of the bacterial host. Phages that rely on this replication strategy exclusively are termed obligately lytic (OL) andare generally regarded as the gold standard for biocontrol (9–11). Predation by OL phages imposes substantial fitness costs on susceptible bacteria and therefore frequently selects for resistant subpopulations with absent or modified receptors (12–15). Since phages can use lipopolysaccharide (LPS), capsule components, pili, flagella, adhesins, efflux pumps, and other virulence or antimicrobial resistance determinants as receptors, phage predation can steer bacterial populations toward virulence-attenuated and antimicrobial-sensitized phenotypes (8, 16–25).

*Salmonella* is a major foodborne pathogen responsible for an estimated 150 million gastroenteritis cases annually, more than one-third of which are attributed to the highly prevalent and virulent serovar Enteritidis (26, 27). Although *Salmonella* is most commonly associated with poultry and egg products, outbreaks have been linked to a wide variety of food commodities, including fresh produce, nut products, confectionery items, and other ready-to-eat foods. Critically, contamination can occur at any point in the food production chain, particularly during processing and distribution (28, 29). Effective sanitation is therefore essential for reducing the risk of contamination in these environments, but is increasingly undermined by bacterial persistence and tolerance to commercial disinfectants (30–35). Sanitizer tolerance has also been linked to antimicrobial and metal resistance in *Salmonella* species, suggesting that such populations may present an elevated risk to public health (36, 37). Consequently, novel approaches capable of enhancing sanitizer efficacy are of considerable interest to the food production industry.

Although phage steering toward hypersensitivity to antibiotics has been reported in several bacterial systems, including *Salmonella* (19, 38–40), comparatively little is known about whether phage-driven selection can similarly enhance susceptibility to commercial disinfectants. In this study, we confirmed that the OL *Salmonella* siphovirus SF1 requires an intact O-antigen layer for infection and lytic replication and consequently selects for O-antigen-defective populations that are hypersensitive to several commercial sanitizers. Crucially, SF1 exhibits antibacterial synergy with multiple membrane-acting disinfectants, consistent with the hypothesis that phage-mediated selection for O-antigen-deficient phenotypes increases population-level susceptibility to these sanitizers. Taken together, these findings support the use of bacteriophages not only as bactericidal agents, but also as evolutionary steering tools capable of sensitizing bacterial populations to existing antimicrobial interventions.

## MATERIALS AND METHODS

### Organisms and media

All experiments were conducted using *Salmonella enterica* sv. Enteritidis strain FSL S5-483, for which the whole-genome sequence was obtained from the *Salmonella* Foodborne Syst-OMICS database (41) or single-gene clean deletion mutants thereof (Table S1). Bacteria were incubated statically on TSB 1.5% (wt/vol) agar at 37°C for 16–18 h, and single colonies were cultured in 5 mL tryptic soy broth (TSB; BD Difco, Franklin Lakes, NJ, USA) or Luria-Bertani (LB; Amresco, Solon, OH, USA) broth at 37°C, shaking at 160 rpm for 16–18 h. LB was used for molecular studies, while TSB was used for all other experiments. For long-term storage, strains were maintained at −80°C in TSB supplemented with 12.5% (vol/vol) glycerol.

Bacteriophage SF1, an OL siphovirus previously isolated from cattle feces (42), was stored at 4°C in salt magnesium (SM) buffer (100 mM NaCl, 8 mM MgSO_4_, 50 mM Tris-HCl at pH 7.5, in dH_2_O) and propagated using the double-layer agar method described previously (43, 44). Soft agar lysate containing SF1 was subsequently resuspended in 3 mL of SM buffer, scraped off plates, incubated overnight at room temperature (RT) to release phages, and centrifuged for 20 min at 4,700 *× g*, 4°C, and the resulting supernatant was sterilized using a 0.45 μm MCE filter (Cytiva, Marlborough, MA, USA) and titered using the double-layer agar method. Amplifications were performed using *S*. Enteritidis FSL S5-483 and repeated continuously to maintain phage titers ≥ 10^9^ PFU mL^−1^.

All disinfectants used in this study (Table S2) were maintained at working concentrations, sterilized using 0.45 μm MCE filters (Cytiva), and diluted to required concentrations in TSB.

### Clean deletion mutant construction

Four candidate genes implicated in phage-host interactions were selected for mutant construction (Table S1) based on previous work (15). For mutants constructed in this study, approximately 500 bp regions upstream and downstream of each target gene were amplified by PCR and used to generate in-frame deletion constructs lacking the coding sequence (CDS) using primers listed in Table S3. Deletion constructs for *wcaJ* and *rfaB* were generated by overlap extension PCR as described previously (45), and cloned into the suicide vector pRE112 (Addgene, Watertown, MA, USA) using T4 ligase (New England Biolabs, Ipswich, MA, USA) following overnight incubation at 16°C. The upstream and downstream flanking regions for *dcm* were assembled directly into pRE112 using the NEBuilder HiFi DNA Assembly Master Mix (New England Biolabs) according to the manufacturer’s protocol.

The recombinant plasmids were transformed into chemically competent SM10λpir cells (Addgene) using the CaCl_2_ method described previously (46). Briefly, mid-log phase (OD_600_ ≈ 0.45) cells were washed and resuspended in chilled 0.1 M CaCl_2_; plasmid DNA was added; and cells were subjected to a 45 s heat shock at 42°C, followed by recovery on ice for 2 min. Cells were then combined with 0.45 mL SOC medium, and cultures were incubated at 37°C for 2 h prior to plasmid extraction using a QIAprep Spin Miniprep Kit (Qiagen, Germany).

Purified recombinant pRE112 plasmids were introduced into electrocompetent *S*. Enteritidis FSL S5-483 cells via electroporation. Briefly, mid-log phase (OD_600_ ≈ 0.45) cells were washed and resuspended in chilled 10% (vol/vol) glycerol; 80 μL aliquots were combined with 100–200 ng of plasmid DNA (≤ 3 μL total volume) and transferred to chilled 1 mm electroporation cuvettes (Bio-Rad, Hercules, CA, USA). Electroporation was performed at 2.5 kV for 2.4 ms; 0.9 mL SOC medium was added immediately after pulsing; and cells were recovered at 37°C, 160 rpm for 2 h before being plated on 1.5% (wt/vol) LB agar supplemented with chloramphenicol (25 μg mL^−1^). For counterselection, colonies were inoculated into LB broth lacking NaCl and supplemented with 8% (wt/vol) sucrose and incubated at 37°C for 24–72 h to select for double-crossover events. Cultures were serially diluted and plated every 24 h, and colonies were PCR-screened for successful allelic exchange. Deletion mutants were confirmed via Sanger sequencing and lack of growth on chloramphenicol-supplemented (25 μg mL^−1^) 1.5% (wt/vol) LB agar plates.

### Planktonic killing assays

Planktonic killing (PK) assays were conducted and analyzed as described previously (47), with minor modifications. Briefly, 0.1 mL of bacterial culture adjusted to 10^6^ CFU mL^−1^ in TSB was combined with an equal volume of phage SF1 diluted in TSB to 10^8^ PFU mL^−1^ in a flat-bottomed 96-well plate (Corning, Corning, NY, USA), yielding a starting multiplicity of infection (MOI) of 100. Plates were incubated in a SpectraMax M2/M2e microplate spectrophotometer (Molecular Devices, Sunnyvale, CA, USA) at 37°C for 20 h, with orbital shaking prior to each reading, and OD_600_ readings were collected automatically every 30 min.

Output data were analyzed using the growth reduction coefficient (GRC) method described previously (47). Briefly, the area (*A_x_*) under the OD_600_ vs time curve for treatment with phage x was computed using the trapezoid approximation method:


$$A_{x}=\int_{t_{i}}^{t_{f}}{OD}_{600}\left(x\right)dt$$

Outputs were then mathematically transformed through comparison with the area (*A*_0_) under the OD_600_ vs. time curve for the untreated strain to yield the GRC for phage *x* on that strain:


$${GRC}_{x}=1-\;\frac{A_{x}}{A_{0}}$$

### Adsorption assays

Adsorption assays were performed as described previously (48), with minor modifications. Bacterial cultures were diluted to 10^8^ CFU mL^−1^ in TSB, and phage SF1 was added at a starting MOI of 5. Samples were collected immediately following phage addition (*t*_0_), and then every 10 min for a total of 60 min, and each sample was immediately sterilized using a 0.45 μm MCE filter (Cytiva) to remove bacterial cells. Serially diluted samples were spotted on lawns of *S*. Enteritidis FSL S5-483 to determine unadsorbed (free) phage titers at each timepoint.

### Single-step growth assays

Single-step growth experiments were conducted as described previously (49), with minor modifications. Bacterial cultures were subcultured 1:10 in fresh TSB and grown to ∼10^9^ CFU/mL^−1^. Phage SF1 was added at a starting MOI of 0.01 and allowed to adsorb at RT for 5 min before cultures were centrifuged at 4,700 *× g*, 4°C for 10 min to remove unadsorbed phage particles. Bacterial pellets were resuspended in 10 mL TSB, and samples were collected immediately (*t*_0_), and then every 10 min for a total of 90 min. Samples were serially diluted and enumerated using the double-layer agar method (43, 44).

### Growth inhibition and phage-disinfectant synergy assays

Assays examining growth inhibition by phage SF1 and various concentrations of commercial sanitizers (see Table S2), alone or in combination, were conducted based on methods described previously (50, 51). Briefly, 0.1 mL of bacterial culture diluted in TSB to 10^6^ CFU/mL^−1^ was added to each well in a flat-bottomed 96-well plate (Corning) and combined with 0.05 mL of either sterile TSB or relevant sanitizers diluted in TSB (final concentration ranges described in Table S2) and 0.05 mL of either sterile TSB or phage SF1 diluted in TSB (final MOI = 10). Plates were incubated in a SpectraMax M2/M2e microplate spectrophotometer (Molecular Devices) at 37°C for 20 h, with orbital shaking prior to each reading, and OD_600_ readings were collected automatically every 30 min.

To quantify bacterial growth across the entire experimental period, the areas under OD_600_ vs. time curves were computed using the trapezoid approximation method. Output values were normalized to untreated controls and expressed as percent growth relative to those controls. To quantify antibacterial activity, normalized growth values were transformed into GRCs as described in a previous section. Phage-disinfectant interactions were then categorized as mathematically synergistic, antagonistic, additive, or autonomous using an established classification framework (47).

### Statistical analysis

Details of statistical analyses and sample sizes are provided in figure legends and, where necessary, in experiment-specific methods sections. Bacterial lag time was computed using the gcplyr package (52) in R Studio v4.3.1, while GraphPad Prism v11.0.0 (GraphPad Software, San Diego, CA, USA) was used to conduct all statistical analyses and construct figures. In all analyses, *P* < 0.05 was considered the threshold of statistical significance.

## RESULTS AND DISCUSSION

### The O-antigen is essential for SF1 adsorption and lytic replication

Receptor identification is critical for the development of phage biocontrol, and is particularly salient for strategies exploiting phage steering (7, 11, 13). In previous work, the OL *Salmonella* siphovirus SF1 was shown to select for survivor populations with mutations in *rfbC*, a dTDP-4-dehydrorhamnose 3,5 epimerase essential for O-antigen biosynthesis, and failure of SF1 to infect a Δ*rfbC* clean deletion mutant confirmed that the O-antigen is required for infection (15, 42). However, resistant survivor populations evolved in the context of SF1 predation frequently contained mutations in three other genes, namely, the methyltransferase *dcm*, the exopolysaccharide phosphotransferase *wcaJ*, and the glycosyltransferase *rfaB*, which is required for complete assembly of the LPS outer core, and the potential regulatory or mechanistic contributions of these genes to the SF1-resistant phenotype remain unclear (15).

**Fig 1:**
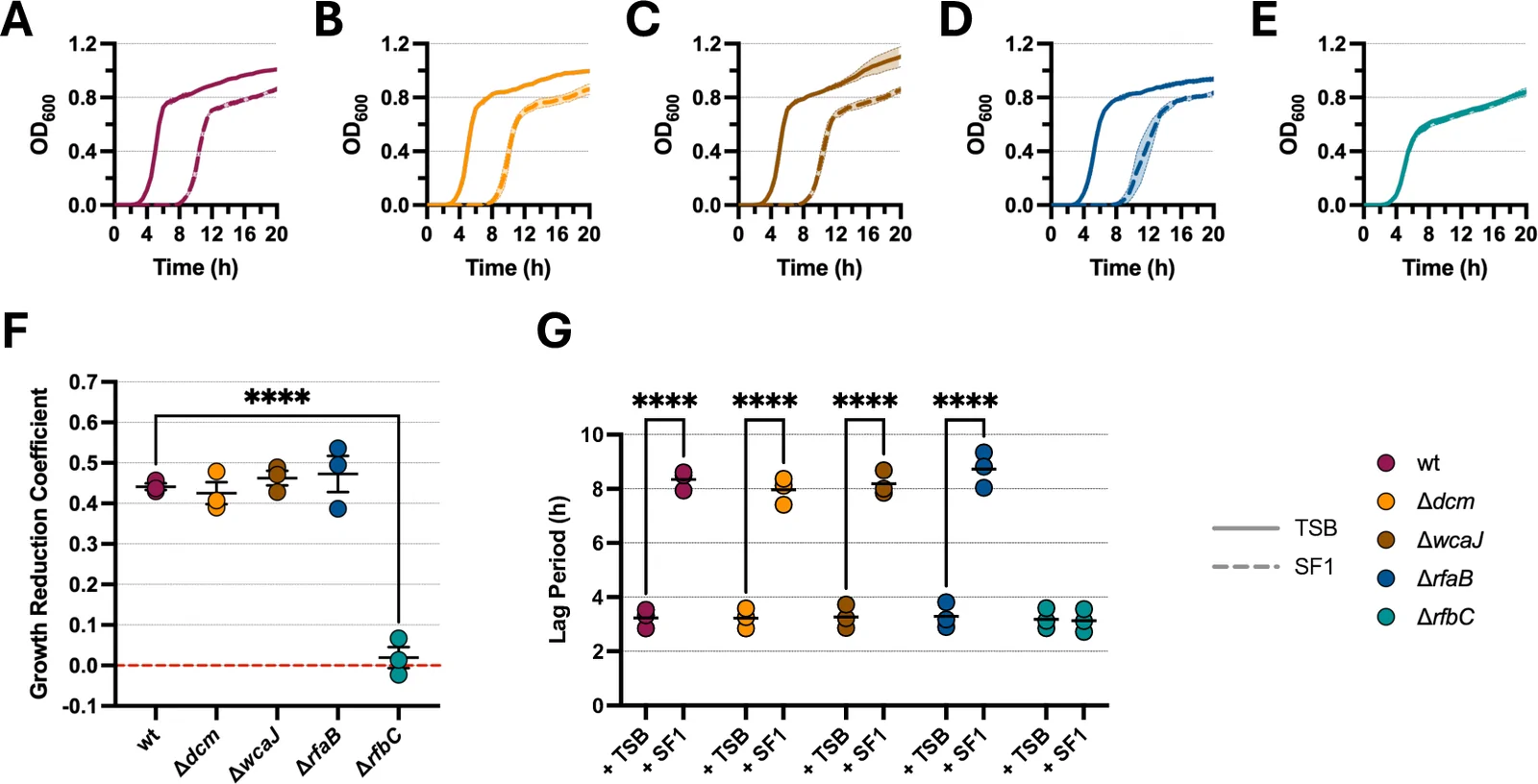
Antibacterial activity by SF1 requires an intact O-antigen layer. Bacterial growth in the absence (solid lines) and presence (dashed lines) of SF1 for *Salmonella* Enteritidis FSL S5-483 wt (**A**), ∆*dcm* (**B**), ∆*wcaJ* (**C**), ∆*rfaB* (**D**), and ∆*rfbC* (**E**). Growth reduction coefficient (GRC) of SF1 on each strain (**F**) and lag period of each strain following co-incubation with TSB or SF1 (**G**). Dashed red line in panel F highlights GRC = 0, which signifies no suppression of bacterial growth. Lines and points represent the means of at least three biological replicates, while shaded regions and error bars show SEM thereof. Differences between groups in panel F were identified using a one-way ANOVA, followed by Tukey’s post-hoc multiple comparison test, while groups in G were compared using Student’s *t*-tests, with **** indicating *P* < 0.0001. For simplicity, only significant differences are shown.

**Fig 2:**
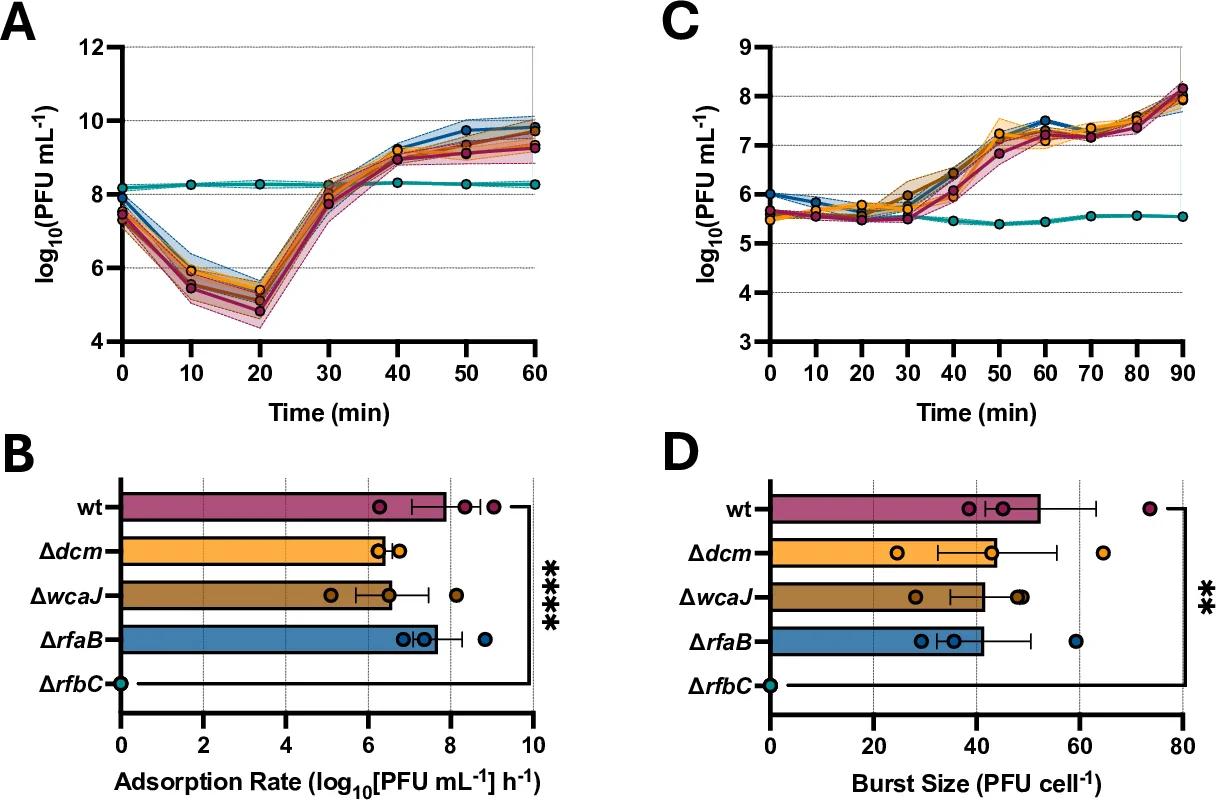
O-antigen is required for SF1 adsorption and replication. Supernatant titer (**A and C**) and computed adsorption rate (**B**) and burst size (**D**) of SF1 co-incubated with *Salmonella* Enteritidis FSL S5-483 wt (maroon), ∆*dcm* (orange), ∆*wcaJ* (brown), ∆*rfaB* (blue), and ∆*rfbC* (teal). Points and bars represent the means of at least three biological replicates, which are shown as points about the mean in bottom panels, while shaded regions and error bars show SEM of those replicates. Differences between groups were identified using one-way ANOVAs, followed by Tukey’s post-hoc multiple comparison tests, with ** and **** indicating *P* < 0.01 and *P* < 0.0001, respectively. For simplicity, only significant differences are shown.

To address this, we procured in-frame deletion mutants of all four genes (Table S1 and S3) in *S*. Enteritidis FSL S5-483 and interrogated their dynamics with SF1. All four mutants grew well in TSB under lab conditions but Δ*rfbC* presented with a growth defect and exhibited a significantly lower overall growth yield when compared to the wt parent strain (*P* < 0.0001; Fig. S1), consistent with findings reported for O-antigen defective mutants in other lineages (22, 25, 53, 54). SF1 modestly reduced the growth of wt FSL S5-483 and mutants Δ*dcm*, Δ*wcaJ*, and Δ*rfaB* over a 20 h period (growth reduction coefficient (GRC) = 0.45 ± 0.05 across all four strains; Fig. 1A through D and F) by delaying the bacterial outgrowth by approximately 6 h (all *P* < 0.0001; Fig. 1G), but this effect was completely abolished in Δ*rfbC* (Fig. 1E and G), on which the GRC of SF1 was reduced to zero (*P* < 0.0001 relative to wt; Fig. 1F). Furthermore, Δ*rfbC* was the only tested strain on which the adsorption rate (Fig. 2A and B; *P* < 0.0001) and burst size (Fig. 2C and D; *P* = 0.0055) of SF1 were reduced to zero, and these properties were not meaningfully altered in the other clean deletion mutants. Taken together, these data confirm that the O-antigen is indispensable for SF1 adsorption and lytic replication in *S*. Enteritidis FSL-S5-483 and imply that while *dcm*, *wcaJ*, and *rfaB* mutations may modulate *rfbC* function when co-occurring with *rfbC* mutations in experimentally evolved populations, these genes play no direct role in conferring resistance to SF1.

These findings are not altogether unexpected since use of the LPS O-antigen as a primary receptor is common among *Caudoviricetes* phages, including those infecting *Salmonella* (16, 19, 39, 55). Crucially, our overall findings in this study may therefore be representative of a broader class of bacteriophages that steer bacterial populations toward LPS-defective phenotypes.

### LPS truncation increases sensitivity to commercial disinfectants

LPS-binding phages have been shown to select for bacterial populations with decreased virulence and increased sensitivity to serum, antimicrobial peptides, and antibiotics (19–22, 39, 40), but steering toward hypersensitivity to commercial disinfectants remains relatively underexplored. In a recent study, Lauman et al. demonstrated that the O-antigen binding *Burkholderia* phage KS12 selects for survivor populations that are hypersensitive to a broad array of membrane-acting antimicrobials, including the cationic sanitizer benzalkonium chloride (BAC). Importantly, this phenomenon was directly linked with O-antigen loss-mediated hyperpermeability of the outer membrane (OM) in these resistant lineages, consistent with the role of the OM as a critical permeability barrier (25, 56–58).

**Fig 3:**
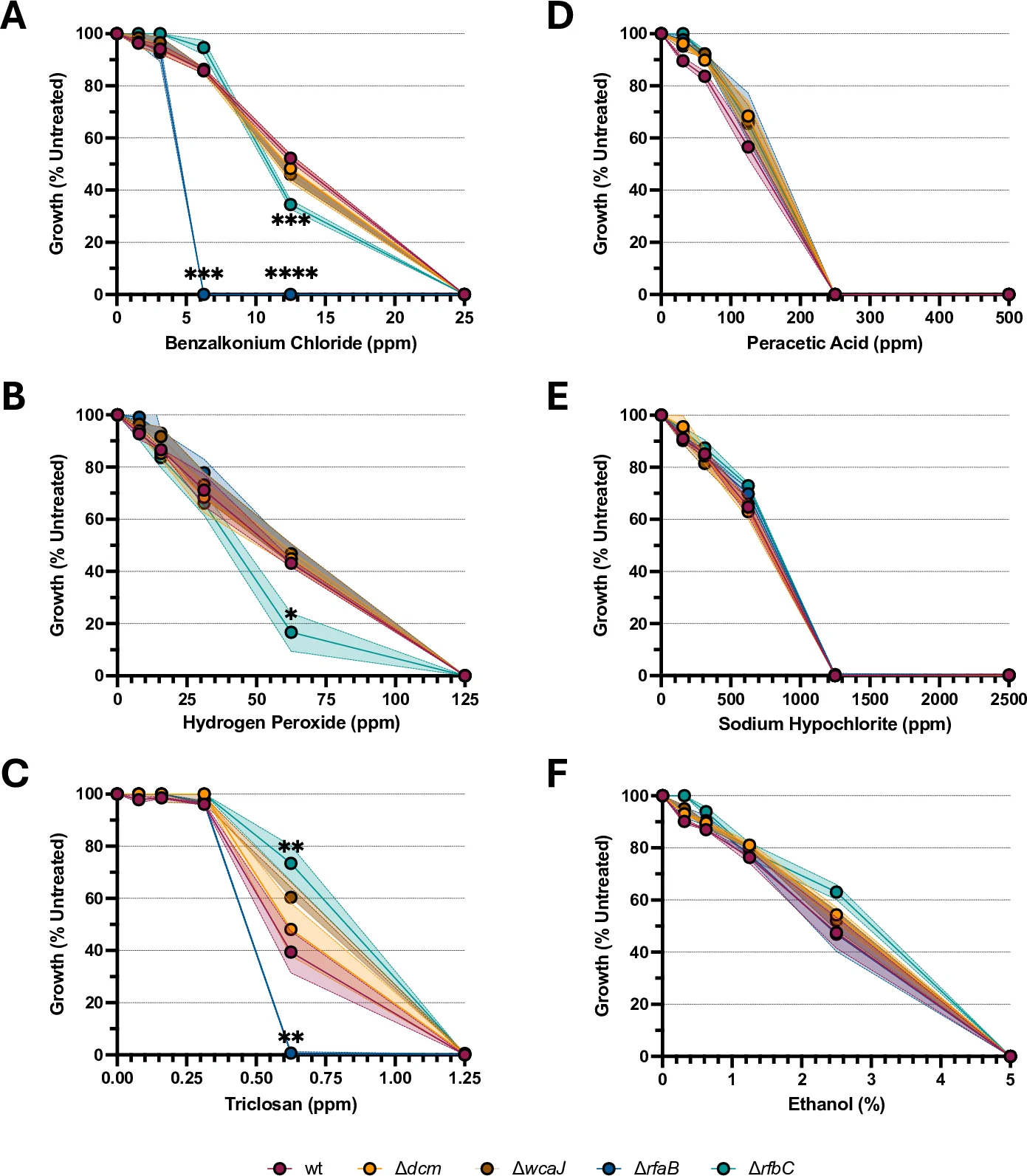
*Salmonella* LPS mutants are hypersensitive to disinfectants. Growth of *Salmonella* Enteritidis FSL S5-483 wt (maroon), ∆*dcm* (orange), ∆*wcaJ* (brown), ∆*rfaB* (blue), and ∆*rfbC* (teal) exposed to various concentrations of benzalkonium chloride (**A**), hydrogen peroxide (**B**), triclosan (**C**), peracetic acid (**D**), sodium hypochlorite (**E**), and ethanol (**F**). Points and shaded regions represent the means and SEM, respectively, of at least three biological replicates. Differences between clean deletion mutants and the wt strain at specific concentrations were identified using one-way ANOVAs, followed by Tukey’s post-hoc multiple comparison tests, with *, **, ***, and **** indicating *P* < 0.05, *P* < 0.01, *P* < 0.001, and *P* < 0.0001, respectively. For simplicity, only significant differences are shown.

In the present study, FSL-S5-483 Δ*rfbC* was observed to exhibit increased sensitivity to BAC (*P* = 0.0007; Fig. 3A) and hydrogen peroxide (H_2_O_2_; *P* = 0.0209; Fig. 3B), and decreased sensitivity to triclosan (*P* = 0.004; Fig. 3C), relative to the wt parent strain. Since BAC is membrane-active, and both H_2_O_2_ and triclosan diffuse into cells across the OM, the efficacy of these three sanitizers may be influenced by changes in OM permeability following O-antigen loss. Simultaneously, however, triclosan is a bacteriostatic compound known to target fatty-acid synthesis and may, therefore, be less effective against slow-growing strains, such as Δ*rfbC*, in which lipid demand is decreased (59, 60). In contrast, the LPS outer core mutant Δ*rfaB* exhibited substantial sensitization to BAC (*P* = 0.0002 and *P* < 0.0001 at 6.25 and 12.5 ppm, respectively; Fig. 3A) and triclosan (*P* = 0.0021; Fig. 3C), but sensitivity to H_2_O_2_ was unaltered (Fig. 3B). Although sensitization to BAC and triclosan is consistent with increased OM permeability in this LPS-truncated but normally growing strain, a satisfying mechanistic explanation for the lack of sensitization to H_2_O_2_ currently remains elusive. FSL-S5-483 Δ*dcm* and Δ*wcaJ* were not meaningfully sensitized to these compounds (Fig. 3A through C), and none of our clean deletion mutants exhibited altered sensitivity to peracetic acid (Fig. 3D), sodium hypochlorite (Fig. 3E), or ethanol (Fig. 3F), implying that the proposed mechanism of steering-mediated sensitization does not apply equally to all disinfectants.

Importantly, the increased overall sensitivity of Δ*rfaB* relative to Δ*rfbC* (Fig. 3A through C) is broadly consistent with the paradigm of rougher LPS mutants generally exhibiting greater sensitivity to antimicrobials (22, 54, 61, 62) and suggests that phages selecting for deeper LPS truncations may induce more pronounced steering-associated trade-offs. Simultaneously, however, the distinct sensitization profiles of these two mutants imply that cocktails composed of both LPS core- and O-antigen-adsorbing phages could increase bacterial sensitivity to a broader range of disinfectants.

### SF1 synergizes with commercial disinfectants

**Fig 4:**
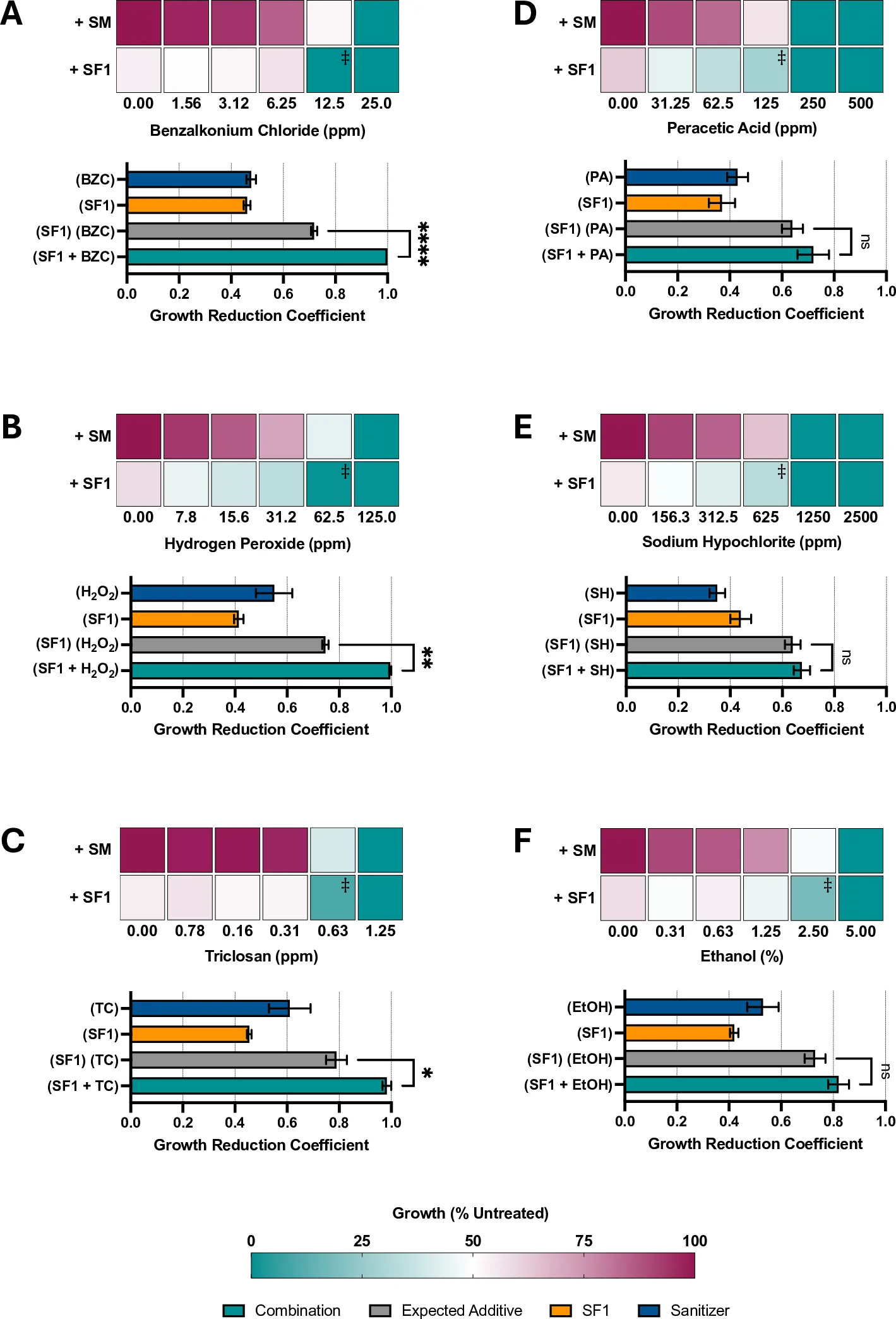
SF1 synergizes with sanitizers. Top panels: normalized growth of *Salmonella* Enteritidis FSL S5-483 exposed to various concentrations of benzalkonium chloride (BAC; **A**), hydrogen peroxide (H_2_O_2_; **B**), triclosan (**C**), peracetic acid (**D**), sodium hypochlorite (**E**), and ethanol (**F**), alone or in combination with SF1. Bottom panels: normalized growth reduction coefficients (GRCs) for treatments with SF1 (orange), relevant sanitizers (blue), and combinations of both agents (teal) at specific conditions identified with dagger (‡) symbols in top panels. Gray bars in bottom panels show the theoretical GRCs expected for combinations that have mathematically additive effects. All presented values are the means of at least three biological replicates, while error bars show SEM thereof. Differences between groups in bottom panels were identified using one-way ANOVAs followed by Tukey’s post-hoc multiple comparison tests, with ns, *, **, and **** indicating *P* > 0.05, *P* < 0.05, *P* < 0.01, and *P* < 0.0001, respectively. For simplicity, only the differences between theoretical (gray) and actual (teal) GRCs of SF1-sanitizer combinations are shown.

To determine whether SF1 might be suitable as an adjuvant to commercial sanitizers, we next tested the antibacterial efficacy of each sanitizer across a range of concentrations in the presence and absence of SF1 against *S*. Enteritidis FSL S5-483. Indeed, SF1 improved the efficacy of all six disinfectants at virtually all tested concentrations (Fig. 4), supporting the use of this phage as an adjuvant. These findings are particularly encouraging in the context of SF1, which exhibits only a modest antibacterial effect when deployed alone (Fig. 1), and add to a growing body of literature suggesting that phages may be therapeutically useful as adjuvants, even when their direct bactericidal activity is suboptimal (47, 51, 63–65).

Importantly, the antibacterial effects of SF1-sanitizer combinations were merely additive for sanitizers to which SF1-selected LPS-mutants Δ*rfaB* and Δ*rfbC* were not sensitized, including peracetic acid (Fig. 4D), sodium hypochlorite (Fig. 4E), and ethanol (Fig. 4F; also see Fig. S2 for growth suppression details), implying that the bactericidal effects of the two agents were independent and did not interact. In contrast, antibacterial effects were found to be mathematically synergistic in combinations containing sanitizers to which at least one of the SF1-selected LPS mutants was hypersensitive, including BAC (*P* < 0.0001; Fig. 4A), H_2_O_2_ (*P* = 0.0023; Fig. 4B), and triclosan (*P* = 0.0486; Fig. 4C). These findings are consistent with the hypothesis that, in addition to its direct bactericidal activity, SF1 potentiates sanitizer efficacy through selection for sanitizer-hypersensitive phenotypes (7, 66). Although similar mechanisms of synergy have previously been demonstrated in combinations wherein phages served as adjuvants to antibiotics, to our knowledge this is the first report demonstrating phage steering as a strategy for deploying bacteriophages as synergizing adjuvants to commercial disinfectants.

### Conclusion

In this study, we confirmed that the OL *Salmonella* phage SF1 requires an intact O-antigen layer for adsorption and lytic replication and demonstrated that O-antigen loss or deeper LPS truncation increases susceptibility to several commercial disinfectants. Importantly, SF1 enhanced the antibacterial activity of all tested sanitizers and furthermore exhibited mathematical synergy with compounds to which SF1-selected LPS mutants were hypersensitive. Collectively, these findings are consistent with the hypothesis that SF1 functions not only as a bactericidal agent, but also as an evolutionary steering tool that enhances sanitizer activity through selection for hypersensitized populations. In practice, phage steering could be incorporated into sanitation strategies by deploying appropriately selected phages alongside or prior to sanitizers, such that direct phage-mediated killing is complemented by selection for surviving populations with increased sanitizer susceptibility.

Although these findings were obtained under controlled conditions using a single phage-host system in well-mixed culture, they nevertheless demonstrate the potential utility of phage steering as a strategy for deploying bacteriophages as adjuvants to commercial disinfectants. Future work should interrogate whether similar “synergy-by-steering” effects occur across diverse phage-host systems and under conditions more representative of food production and processing environments, including on contaminated surfaces and within complex polymicrobial communities. Furthermore, the long-term stability of these steering associated trade-offs, particularly the potential emergence of residual subpopulations tolerant to both phages and sanitizers, warrants investigation in future work. More broadly, our findings highlight the potential value of exploiting evolutionary trade-offs to improve control of bacterial pathogens in the food production chain.

## SUPPLEMENTAL MATERIAL

Supplemental material aem.01437-26-s0001.docx Fig. S1 and S2; Tables S1 to S3.

ASM does not own the copyrights to Supplemental Material that may be linked to, or accessed through, an article. The authors have granted ASM a non-exclusive, world-wide license to publish the Supplemental Material files. Please contact the corresponding author directly for reuse.
