# Supplementary material for: A Salmonella phage steers bacterial populations toward hypersensitivity to benzalkonium chloride, hydrogen peroxide, and triclosan
Source: Appl Environ Microbiol. 2026 Oct 1;92(10):e01437-26. doi: 10.1128/aem.01437-26 (PMC13689483; doi:10.1128/aem.01437-26)
Supplement: Supplementary file 1 [file aem.01437-26-s0001.docx]

**Figure S1: O-antigen loss causes defective growth in Salmonella.** Growth curves (**A**) and normalized area under the curve (AUC; **B**), for Salmonella Enteritidis FSL S5-483 wt (maroon), $\Delta$dcm (orange), $\Delta$wcaJ (brown), $\Delta$rfaB (blue), and $\Delta$rfbC (teal). Lines and points show the means of at least three biological replicates, while shaded regions and error bars show SEM thereof. Differences between groups in **B** were identified using a one-way ANOVAs followed by Tukey’s Post-Hoc multiple comparisons tests, with **** indicating p < 0.000. For simplicity, only significant differences are shown.

**Figure S2: SF1 synergizes with sanitizers.** Growth curves for Salmonella Enteritidis FSL S5-483 exposed to TSB (maroon), various sanitizers (blue) including Benzalkonium Chloride (**A**), Hydrogen Peroxide (**B**), Triclosan (**C**), Peracetic Acid (**D**), Sodium Hypochlorite (**E**), and Ethanol (**F**), SF1 (orange), and combinations of both (teal), at specific conditions identified with dagger ($\ddagger$) symbols in **Fig. 4**. Lines and shaded regions show the means and SEM, respectively, of at least three biological replicates.

**Table S1:** Bacterial strains used in this study.

| Strain | Characteristics | | Reference |
| --- | --- | --- | --- |
|  | **Affected Protein** | **Affected Function** |  |
| FSL S5-483 wt |  |  | (1) |
| FSL S5-483 $\boldsymbol{\Delta}$*dcm* | DNA cytosine methyltransferase | Gene regulation | This study. |
| FSL S5-483 $\boldsymbol{\Delta}$*wcaJ* | Undecaprenyl phosphate glucose phosphotransferase | Exopolysaccharide biosynthesis | This study. |
| FSL S5-483 $\boldsymbol{\Delta}$*rfaB* | glycosyltransferase II | LPS outer core assembly | This study. |
| FSL S5-483 $\boldsymbol{\Delta}$*rfbC* | dTDP-4-dehydrorhamnose 3,5 epimerase | O-antigen biosynthesis | (2) |

**Table S2:** Disinfectants used in this study.

| Sanitizer | Class / Type | Mechanism of Action | Concentration Range |
| --- | --- | --- | --- |
| Benzalkonium Chloride | Cationic surfactant | Membrane disruption | 0.1 – 50 ppm |
| Hydrogen Peroxide | ROS oxidizer | DNA & protein damage | 1 – 500 ppm |
| Triclosan | FabI inhibitor | Blocks fatty acid synthesis | 0.5 – 250 ppm |
| Peracetic Acid | Strong oxidizer | Protein oxidation | 4 – 2000 ppm |
| Sodium Hypochlorite | Chlorine oxidizer | DNA & protein chlorination | 5 – 2500 ppm |
| Ethanol | Alcohol | Membrane disruption & protein denaturation | 0.02 – 12.5% |

**Table S3:** Details of mutant construction in this study

| Gene / primer | Sequence | Method |
| --- | --- | --- |
|  |  |  |
| *dcm* |  | NEBuilder Hifi Assembly |
| Forward primer (1) 5’ $\boldsymbol{\to}$ 3’ | CATGAATTCCCGGGAGAGCTGTACCTGTTTCCCGCCAG |  |
| Reverse primer (1) 5’ $\boldsymbol{\to}$ 3’ | GGTTAAATGTAGCCACGCGCAGTAAAAATATG |  |
| Forward primer (2) 5’ $\boldsymbol{\to}$ 3’ | GCGCGTGGCTACATTTAACCGGCCAAAAATAATTG |  |
| Reverse primer (2) 5’ $\boldsymbol{\to}$ 3’ | CAAGCTTCTTCTAGAGGTACCCTGACTTTCCGGCAGAAAAAC |  |
|  |  |  |
| *wcaJ* |  | Overlap extension PCR (REs: SacI; KpnI) |
| Forward primer (1) 5’ $\boldsymbol{\to}$ 3’ | GCGAGCTCGCGCGCATCACTATTTTCGTG |  |
| Reverse primer (1) 5’ $\boldsymbol{\to}$ 3’ | GGTCAGGTTCCGTTGTTCCTGTG |  |
| Forward primer (2) 5’ $\boldsymbol{\to}$ 3’ | AGGAACAACGGAACCTGACCCA |  |
| Reverse primer (2) 5’ $\boldsymbol{\to}$ 3’ | GAGGTACCATCGCTCAGCCAAAAGACCAC |  |
|  |  |  |
| *rfaB* |  | Overlap extension PCR (REs: SacI; KpnI) |
| Forward primer (1) 5’ $\boldsymbol{\to}$ 3’ | GCGAGCTCTCGCTGGCGTGACAAAGATT |  |
| Reverse primer (1) 5’ $\boldsymbol{\to}$ 3’ | TTCTGCTCATAGTGAATATACCCCAGAGT |  |
| Forward primer (2) 5’ $\boldsymbol{\to}$ 3’ | AACTCTGGGGTATATTCACTATGAGCAGAA |  |
| Reverse primer (2) 5’ $\boldsymbol{\to}$ 3’ | GCGGTACCGATTTTAAGCGCTCACAGTCGATTAGG |  |
|  |  |  |
| *rfbC* |  | KO strain obtained from Chen et al. (2) |

**References**

1.	Emond-Rheault JG, et al. 2017. A Syst-OMICS approach to ensuring food safety and reducing the economic burden of salmonellosis. Front Microbiol 8.

2.	Chen L, Zhao X, Wongso S, Lin Z, Wang S. 2024. Trade-offs between receptor modification and fitness drive host-bacteriophage co-evolution leading to phage extinction or co-existence. ISME Journal 18.
